# Recent Advances in Phytogenic Feed Additives in Poultry Nutrition: Mechanistic Insights, a Gulf Region Perspective, and Future Directions

**DOI:** 10.3390/vetsci13101035

**Published:** 2026-09-30

**Authors:** Hanan Al-Khalaifah, Sheikh Shreaz, Tahani Al-Surrayai

**Affiliations:** Food Security Program, Environment and Life Sciences Research Center, Kuwait Institute for Scientific Research, Kuwait City 13109, Kuwait

**Keywords:** phytogenic feed additives, poultry nutrition, gut microbiota, sustainable poultry production

## Abstract

Phytogenic feed additives (PFAs) have attracted growing interest as plant-derived components of antibiotic-reduction strategies in poultry nutrition. Reported effects include modulation of the intestinal microbiota, digestive function, oxidative status, immune responses, and product quality; however, both the magnitude and direction of these effects vary with botanical source, chemotype, formulation, dose, basal diet, bird type, and environmental conditions. This narrative review evaluates the functional roles and molecular mechanisms of PFAs, formulation and delivery technologies, omics-based approaches, safety and regulatory considerations, and key evidence gaps. A dedicated Kuwait and Gulf-region perspective is incorporated to consider how heat stress, water and feed constraints, and local botanical resources may shape research priorities and commercial translation. Regional observations are clearly distinguished from evidence obtained in general poultry studies. Future research should prioritize standardized experimental frameworks, quantitative dose-response relationships, direct measurements of intestinal delivery and bioavailability, long-term commercial validation, and appropriately validated artificial-intelligence tools for precision nutrition.

## Simple Summary

Phytogenic feed additives (PFAs) are plant-derived ingredients incorporated into poultry diets to support bird health and production. This review examines their principal phytochemical classes and the mechanisms through which they influence the gut microbiota, digestive function, redox regulation, immunity, and intestinal barrier integrity. It also addresses formulation technologies, omics approaches, and safety considerations. The available evidence is heterogeneous, reflecting differences among studies in botanical composition, extraction methods, inclusion rates, basal diets, bird type, and environmental conditions. A dedicated Kuwait and Gulf-region perspective is included, recognizing that heat stress and other production constraints may affect both the performance and the practical deployment of PFAs. This regional discussion is presented as context-specific and is not intended as evidence that can be automatically generalized to all poultry systems. Future research should prioritize standardized preparations, dose-response trials, quantitative validation of intestinal delivery and bioavailability, long-term studies, and carefully validated data-driven tools for precision nutrition.

## 1. Introduction

The world poultry sector has gone through a significant paradigm shift due to the growing limitations on the routine prophylactic use of antibiotic growth promoters (AGPs). Antibiotics were extensively used in poultry feed for decades to achieve higher feed efficiency, control of bacterial diseases, and rapid growth [1]. Nonetheless, increasing doubts about the resistance of antimicrobials and the risk that animals are capable of acquiring such resistance and passing it to people have led regulatory agencies and research professionals to re-evaluate their daily application in the animal production system. Due to these developments, regulatory frameworks globally have shifted, although the extent and nature of these restrictions differ considerably among jurisdictions. It is critical to distinguish between legislative bans on sub-therapeutic antibiotics used strictly for growth promotion (zootechnical AGPs) and the regulated use of antimicrobials for veterinary disease management. For example, the European Union has implemented a comprehensive ban on zootechnical AGPs, whereas jurisdictions following United States Food and Drug Administration (FDA) guidelines have phased out the use of medically important antibiotics for growth promotion while continuing to regulate their targeted prophylactic and therapeutic applications under veterinary oversight for the treatment of clinical disease [2]. These changes have posed challenges to maintaining productivity and flock health, particularly by increasing the risk of enteric diseases, flock mortality, and reduced feed conversion efficiency. Phytogenic feed additives (PFAs) may help address some of these industry-wide challenges by providing antimicrobial activity, enhancing intestinal barrier integrity, and supporting local immune responses without the direct selection pressure associated with antibiotic use. Recent scientific studies point out the large spectrum of possible alternatives such as probiotics, prebiotics, organic acids, and phytogenic compounds [3]. While probiotics and organic acids primarily modulate gut microbiota and intestinal pH, PFAs may offer additional benefits through their antioxidant and anti-inflammatory activities, effects on feed palatability, and potential stimulation of endogenous digestive enzyme secretion [4]. The changes have resulted in an ever-growing body of literature dedicated to finding effective methods of non-antibiotic production of poultry [1]. Initial research on such options focused on the necessity of combined nutrition methods that can enhance the health of the gut and metabolic efficiency in birds [5]. Plant-based feed additives have received specific interest among the various options suggested due to their natural source and their various biological functions [6].

In this framework, PFAs have gained prominence as one of the most promising nutritional interventions in contemporary poultry production. PFAs are natural bioactive compounds that are based on herbs, spices, plant extracts, as well as essential oils (EOs) that are included in poultry diets to boost their performance and health. The scientific understanding of such plant-based additives has changed a lot in the last twenty years, as it now represents a more complex form of herbal supplementation consisting of specifically designed phytogenic blends that possess specific functional characteristics. According to many studies, these compounds can cause stimulation of the digestive secretions, enhance the absorption of nutrients, and possess antimicrobial and antioxidant effects in the gastrointestinal tract. Due to all these complex impacts, PFAs have become a promising alternative to AGPs in poultry diets [7]. However, it is crucial to recognize that the efficacy of PFAs is highly heterogeneous. Their biological effects depend strongly on the botanical source, chemotype, extraction procedure, active compound concentration, and environmental conditions. Consequently, experimental outcomes can vary, and the literature frequently reports null, inconsistent, or strictly dose-dependent findings that must be critically evaluated [4,8]. As innovative and extensive reviews show, the widespread use of phytogenic additives in commercial poultry feeds is also increasing as producers are finding sustainable nutritional solutions that cater to consumer demands regarding the use of antibiotics in animal feeds [3]. Studies on popular plant substances like garlic and turmeric have also shown that they can be used as a tool to improve growth performance and immune status when added to the broiler feed [9]. Equally, experimental studies have documented that a phytogenic supplement of turmeric has the potential to deliver performance enhancement equal to certain traditional feed supplements utilized in poultry production [10]. All these findings reflect the growing role of phytogenic strategies within the larger trend toward the use of natural feed additives.

Recent advances in phytogenic research are not solely about the amount of herbs or plant powders that can be added to the poultry meals. Modern research tends to pay more attention to the identification of particular bioactive compounds, their mechanisms of action, and the optimal way of their delivery in the feed. The initial studies on the subject of EOs emphasized their antimicrobial effects and the possibility of affecting digestion among chickens [11]. Based on this, more recent research has investigated the interaction between EO constituents and gut microbiota, digestive enzymes, and host immune pathways to enhance the overall performance of the bird [12]. Besides EOs, numerous botanical extracts are actively being considered for use in the prevention of diseases and therapeutic support in flocks of poultry [13]. Nutritional, physiological, and molecular biological advancements also enabled researchers to investigate the effects of phytogenic compounds on gene expression and metabolic control in birds and gain a greater understanding of their functionality [14].

Recent experimental studies have shown that phytogenic blends can influence intestinal morphology, immune responses, growth performance, carcass characteristics, and meat quality in poultry [15,16]. These findings have contributed to a shift from the traditional descriptive use of herbs toward more mechanistic and applied approaches that integrate animal nutrition, microbiology, analytical chemistry, and molecular biology. Increasing attention to specific bioactive compounds and standardized formulations further highlights the importance of understanding how the source material, dosage, delivery system, and experimental conditions influence the biological responses to PFAs [17,18,19].

Despite the expanding body of literature, important knowledge gaps remain regarding the molecular pathways through which PFAs exert their effects, the reproducibility of dose-response relationships, and the consistency of their biological effects under different environmental and production conditions [20]. Therefore, this review integrates phytochemical classification with current evidence on mechanisms of action, formulation and delivery technologies, multi-omics approaches, and safety and regulatory considerations. Particular emphasis is placed on distinguishing findings supported by general poultry research from observations that are specific to particular production environments. The review also considers region-specific challenges, including environmental stressors relevant to poultry production in arid and hot climates, while recognizing that such context-specific evidence requires appropriate experimental validation.

### Literature Search Strategy

To ensure a comprehensive evaluation and justify the focus on recent advances, a narrative literature search was conducted using primary scientific databases, including PubMed, Scopus, and Web of Science. The search targeted literature published predominantly between 2015 and 2026, while foundational studies were retained where necessary for context. Search strings included combinations of keywords such as “phytogenic feed additives”, “poultry nutrition”, “gut microbiota”, “heat stress”, and “omics technologies”. During the selection process, priority was given to poultry-specific in vivo experimental studies, comprehensive meta-analyses, and systematic reviews to ensure the reliability and relevance of the synthesized evidence.

## 2. Diversity and Functional Classification of Phytogenic Feed Additives

PFAs are natural botanical preparations and materials used in poultry diets to improve productivity, gastrointestinal health, and immune function. These preparations include herbs, plant extracts, EOs, and oleoresins, which contain various bioactive phytochemicals, including polyphenols, terpenoids, and organosulfur compounds [4,20]. PFAs are also employed in the production of chickens, as they are natural alternatives to AGPs. Rather than exerting nonspecific effects, these compounds may act through several molecular mechanisms, including targeted antimicrobial activity through disruption of microbial cell membranes, cellular protection through direct scavenging of reactive oxygen species (ROS), and enhanced nutrient utilization through stimulation of endogenous digestive enzyme secretion [4,21]. PFAs may be categorized depending on their physical type and the character of the plant-based compounds. The major categories, classified primarily according to their physical formulation rather than taxonomic classification, are summarized in Table 1, which illustrates the diversity of plant-derived additives used in poultry nutrition. As shown in Table 1, the functional outcomes in poultry, ranging from immune modulation to improvements in meat quality, are not uniformly distributed but depend largely on the specific classes and concentrations of active compounds, such as curcuminoids and capsaicinoids, present in these different formulations [20]. Their biological activity depends largely on the concentration and type of bioactive compounds present in the plant material. Understanding these forms provides a foundation for examining the phytochemical composition and functional mechanisms of PFAs discussed in the following sections.

### 2.1. Structural Forms of Phytogenic Feed Additives

PFAs comprise a diverse group of plant-derived substances incorporated into poultry diets to support productivity, gastrointestinal health, and physiological functions. They are commonly classified according to their physical form and functional characteristics into herbs, plant extracts, EOs, and oleoresins [44]. Herbs generally consist of dried plant materials, including leaves, seeds, roots, or bark, that are incorporated directly into feed. Plant extracts are more concentrated preparations obtained through extraction procedures designed to enrich specific bioactive constituents [17]. EOs are volatile aromatic fractions of plants, typically obtained by steam distillation, whereas oleoresins are semi-solid extracts containing both volatile and non-volatile constituents [45,46]. These forms differ in their physicochemical characteristics, stability, and biological activity. For example, crude plant materials may be more susceptible to degradation during feed processing, whereas encapsulated EOs and concentrated extracts may provide greater protection against thermal degradation during pelleting and facilitate controlled release within the gastrointestinal tract [47,48]. Claims of increased systemic bioavailability, however, should be restricted to formulations supported by direct pharmacokinetic or tissue-exposure measurements.

EOs have been widely investigated for their potential antimicrobial, antioxidant, and digestive-modulating properties and may influence feed intake and gastrointestinal function in broiler chickens [49,50]. Similarly, phytogenic formulations containing EOs have been associated with changes in growth performance, immune-organ development, and carcass characteristics in broilers [51]. Among specific botanical additives, garlic and ginger are notable examples whose biological effects have been linked to characteristic bioactive constituents. In garlic, organosulfur compounds, including allicin, and in ginger, pungent phenolic compounds such as gingerols, have been investigated for their potential roles in modulating digestive and productive responses in poultry [31].

### 2.2. Phytochemical Diversity in Phytogenic Feed Additives

The diversity of phytochemicals present in plant materials is an important determinant of PFA activity. These compounds include phenolic constituents, such as flavonoids and tannins; terpenoids, which comprise many EO constituents; alkaloids; and organosulfur compounds [4,52,53]. Their chemical properties influence their biological activities and mechanisms of action, while factors such as plant species, growing conditions, extraction procedures, storage, and formulation can substantially modify the resulting phytochemical profile. Importantly, the biological effects of PFAs are not consistently positive; dose-dependent, null, and variable responses have been reported, and inappropriate inclusion levels may adversely affect palatability or contribute to antinutritional effects [20,54]. Accordingly, the following classification focuses on broadly investigated phytochemical groups rather than their geographic distribution or availability.

#### 2.2.1. Phenolics and Polyphenols

These are considered one of the most significant groups of phytochemicals in plant-based feed additives. This category can contain flavonoids, tannins, phenolic acids, and lignans, which have high antioxidant capacity and could counteract free radicals and alleviate oxidative stress in living organisms [55]. Dietary antioxidants are important for physiological stability because oxidative stress may affect growth and immune systems in poultry adversely. Polyphenolic compounds have been shown to stimulate the antioxidant defense system and boost immune response in broiler chickens [56,57]. Comparative analysis of tannins from various plant sources found a positive influence on growth performance, immune condition, and intestinal microbial balance when tannins were added to the poultry feed [58]. However, it is critical to note the dose-dependent nature of these compounds. While moderate levels may provide antioxidant and antimicrobial benefits, higher concentrations of tannins can exert anti-nutritional effects by precipitating dietary proteins and reducing nutrient digestibility [59]. To a great extent, these advantages can be explained by the capacity of polyphenols to control oxidative effects and improve the integrity of the intestinal barriers. Moreover, most of the plant extracts that contain a high phenolic content have been reported to enhance antioxidant status and production, especially among aged laying hens [60]. The results are indicative of the idea that polyphenols also play a major role in cellular stability, metabolic efficiency, and in general poultry health.

#### 2.2.2. Terpenoids and Essential Oils

Another important group of phytochemicals comprises terpenoids, which are prevalent in aromatic plants and EOs. Other examples of compounds that add flavor to numerous herbs and spices and exhibit high antimicrobial activity include thymol, carvacrol, menthol, and eugenol [61]. Their mechanism of action mainly involves interfering with microbial cell membranes, thus preventing the proliferation of the pathogenic microorganisms of the gastrointestinal tract [62]. Digestion and nutrient absorption in poultry nutrition have been linked to terpenoid nutrients supplied as EOs because the terpenoids have been shown to stimulate the secretion of digestive enzymes. The compounds are also used to control the intestinal microbial populations, which results in better gut health and feed efficiency [63]. Consequently, EOs comprising a high content of terpenoids are often used as part of phytogenic feeds to improve the performance, immunity, expression of digestive enzymes, and health of poultry [64]. Other effects of terpenoids, in addition to antimicrobial activity, are antioxidant and anti-inflammatory effects that can potentially protect intestinal tissues and decrease physiological stress [21,65]. Due to their versatile biological functions, these compounds are valuable functional ingredients of phytogenic additives in the nutrition of poultry. Similar to polyphenols, the biological efficacy of EOs is highly dose-dependent. Excessive inclusion rates may negatively affect feed palatability, induce toxicity, and disrupt gut homeostasis [61].

#### 2.2.3. Alkaloids and Organosulfur Compounds

Alkaloids and organosulfur compounds are other bioactive components that are prevalent in a number of medicinal plants that serve as phytogenic feed additives. Alkaloids consist of nitrogen-based compounds that are characterized by antimicrobial and anti-inflammatory effects [66]. Because alkaloids can possess potent pharmacological activities, establishing appropriate dose-related safety margins is essential to minimize the risk of toxicity and metabolic disturbances in poultry [67]. They are found at relatively low levels in most feed plants; however, they are the most important in the biological activity of phytogenic formulations. Organosulfur compounds are also found in large amounts in plants of the family *Allium*, e.g., garlic and onions. Allicin and diallyl sulfide compounds have good antimicrobial, antioxidant, and immunomodulatory properties [32]. Food supplementation based on compounds in garlic has been demonstrated to enhance performance in poultry, boost immunity, and have a beneficial effect on gut microbial flora [68]. These bioactive molecules have the effect of promoting the health of the intestine by maintaining the balance of the microbes and promoting metabolism. As a result, alkaloids and organosulfur compounds play a major role in the practical value of the plant-based additives to poultry food.

### 2.3. Extraction Technologies and Analytical Characterization

The extraction methods have been enhanced, which makes the recovery of bioactive phytochemicals in plant materials that are utilized in poultry nutrition better. Traditional processes (steam distillation and solvent extraction) are still common in the extraction of oils and extracts of plants [69]. Nevertheless, recent methods such as supercritical fluid extraction, microwave-assisted extraction, and ultrasound-assisted extraction have improved the rate of extraction and, at the same time, maintained the biological activity of phytogenic compounds [70]. The development of better ways of analysis has also made it possible to accurately characterize compounds created by plants. High-performance liquid chromatography and gas chromatography-mass spectrometry are methods that can be used to identify and determine the concentration of phytochemicals in PFAs [71]. Such analysis methods can be used to learn more about the chemical composition of plant extracts and guarantee product consistency. The effective use of phytogenic additives in the process of animal nutrition requires quality assurance and standardization. While advanced extraction methods can maximize the recovery of bioactive compounds, rigorous analytical characterization is essential for reproducibility. Quantitative standardization using specific marker compounds can help monitor batch-to-batch variability and ensure consistent levels of selected active compounds, even when plant sources or processing conditions vary [72]. Research on phytogenic additives in monogastric animals highlights that better extraction systems and stringent quality control systems are required to guarantee uniform performance and stability in livestock production [73].

### 2.4. Challenges in Phytochemical Variability and Standardization

PFAs have a number of issues associated with standardization and variability despite their increased application in the nutrition of poultry. Comparability among experiments is influenced by multiple heterogeneous factors. Specifically, botanical identity, chemotype, geographical origin, harvest stage, extraction solvent, storage conditions, feed processing conditions, such as pelleting temperature, and the absence of analytical normalization can significantly alter the final chemical profile [74]. These factors can result in uneven concentrations of bioactive compounds, and this can influence the biological activity and end performance of phytogenic additives. Moreover, the variations in extraction and formulation strategies may add to the variations in the quality and effectiveness of products [75]. According to reviews on PFAs, appropriate standardization of raw materials, extraction methods, and dosage should be used to obtain reliable outcomes in the production of poultry [4,76]. To achieve this level of standardization in practice, the industry should transition from uncharacterized crude extracts toward standardized formulations quantified using high-performance liquid chromatography (HPLC) for specific marker compounds. For example, formulations could be standardized according to the concentration of thymol in thyme or oregano extracts. Furthermore, standardization efforts should be coupled with technological approaches such as microencapsulation and lipid matrix embedding to protect volatile phytoconstituents during high-temperature feed pelleting and support consistent intestinal delivery. A resolution to these issues by means of better quality control and uniform production processes will be key to maximizing the advantages of phytogenic additives in modern poultry systems.

## 3. Molecular and Physiological Mechanisms of Phytogenic Feed Additives in Poultry

**Figure 1:**
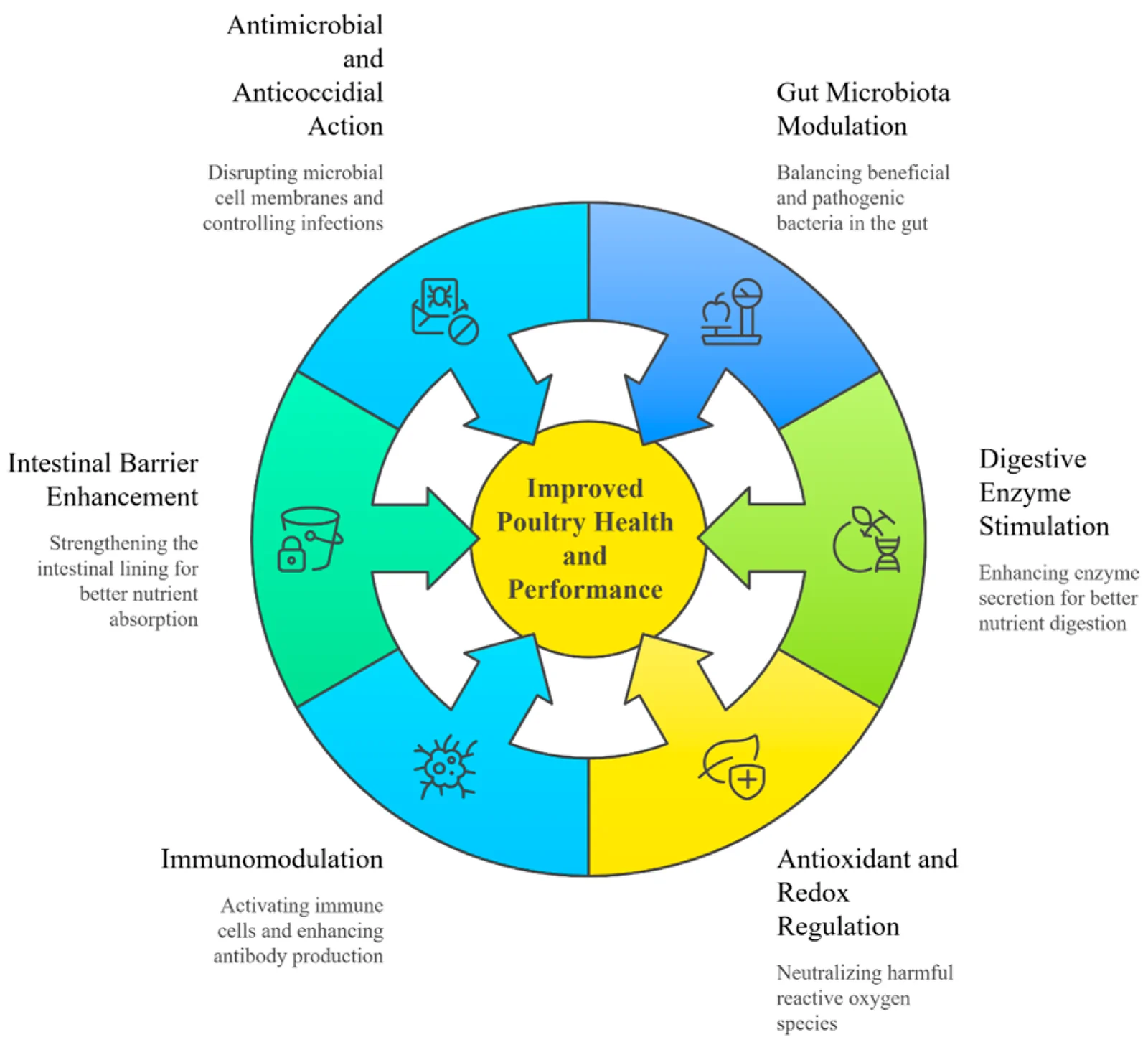
Mechanisms of action of PFAs in poultry nutrition, illustrating their multi-target biological functions including gut microbiota modulation, stimulation of digestive enzyme activity, antioxidant and redox regulation, immunomodulation, enhancement of intestinal barrier integrity, and antimicrobial and anticoccidial effects that collectively contribute to improved poultry health, nutrient utilization, and production performance [8,77,78].

PFAs have multiple biological impacts on poultry that combine to increase intestinal health, metabolic efficiency, and immune competence. These natural plant-based substances react with digestive, microbial, and physiological metabolisms, leading to better growth performance and general bird production. The most important processes by which phytogenic additives can affect the health of poultry are mentioned in Figure 1. It is crucial to distinguish direct mechanisms supported by experimental evidence, such as direct radical scavenging and microbial membrane disruption, from downstream interconnected physiological outcomes, such as immune signaling and epithelial integrity. Rather than acting through independent pathways, these mechanisms may interact to support intestinal function and maintain physiological balance in poultry production systems [8,77,78].

### 3.1. Microbiota-Mediated Effects of Phytogenic Compounds

As per the available reports, PFAs are also beneficial in the development of the intestinal microbial ecosystem of poultry. Phenolics, flavonoids, and EOs, which are bioactive plant compounds, have the ability to inhibit the growth of hazardous microorganisms and to stimulate the growth of the beneficial microorganisms [79]. These antimicrobial effects may help maintain a balanced intestinal microbiota and reduce the risk of enteric microbial infections [21]. At the cellular level, these effects may involve modulation of receptor-mediated signaling, such as regulation of toll-like receptors, and provision of substrates that support short-chain fatty acid (SCFA) production pathways, which may contribute to the maintenance of intestinal barrier integrity [80]. However, microbiota responses are highly dependent on compound dose, basal diet, and the specific intestinal compartment [81]. Furthermore, the resolution of available evidence varies considerably according to the analytical methods employed. While foundational studies relied primarily on conventional culture methods and targeted bacterial quantification, contemporary mechanistic investigations increasingly use high-resolution 16S rRNA sequencing and shotgun metagenomics to characterize changes in microbial communities [82]. Furthermore, phytogenic compounds can also affect microbial fermentation and gut metabolic processes and pathways overall, enhancing gut stability and total nutrient use [4]. The stability of microbes is necessary to ensure the health and disease prevention of the intestine in poultry production systems. Phytogenic additives have been reviewed and analyzed, and phytogenic compounds have the potential to regulate the microbial community and improve gastrointestinal functions in the avian diet [83]. Moreover, phytobiotic compounds are identified as natural agents that enhance gut microbial ecology as well as decrease pathogen colonization in poultry production [77].

### 3.2. Phytogenic Enhancement of Digestive Function and Nutrient Utilization

It has been established that phytogenic additives can increase the efficiency of digestion by enhancing the secretion of enzymes and their action. Specific bioactive compounds, such as piperine from black pepper and cinnamaldehyde from cinnamon, may enhance the activity of digestive enzymes such as proteases, lipases, and amylases, thereby supporting the digestion of dietary proteins, fats, and carbohydrates [4,21]. These effects may involve transcriptional regulation of digestive enzyme genes, stimulation of pancreatic secretions, and modulation of brush border membrane nutrient transporters, thereby contributing to nutrient utilization. Increased enzyme activity increases the availability of nutrients and the efficiency of feeds in poultry. There are also specific phytochemicals, such as curcumin, which may also affect the production and secretion of bile and other digestive secretions that facilitate the digestion and absorption of fats [4]. All these effects are used together to enhance growth performance and feed ratio. The initial studies of the herbal feed supplements have stated that they may improve food digestion and metabolic rate in the nutrition of animals [84]. According to more recent reviews, phytogenic compounds may also be used as natural growth promoters because they enhance digestive physiology and nutrient absorption in poultry [78].

### 3.3. Phytogenic Regulation of Antioxidant Defense and Redox Homeostasis

The phytogenic feed supplements contain high levels of antioxidant compounds that can balance oxidative stress in the tissues of poultry [85,86]. It is important to distinguish between direct radical scavenging effects and the activation of endogenous antioxidant pathways. Directly, reactive oxygen species can be neutralized by hydroxyl groups present in polyphenols, flavonoids, and other phytochemicals [87]. Indirectly, as previously noted, specific phytochemicals can activate endogenous antioxidant defense systems through the *Nrf2* signaling pathway, leading to the upregulation of antioxidant enzymes such as superoxide dismutase (SOD), catalase (CAT), and glutathione peroxidase (GSH Px) [88,89]. Redox homeostasis is critical in the maintenance of metabolic efficiency, immunological competence, and effective performance in poultry [90]. In a meta-analysis of phytogenic extracts as part of poultry diets, it was proven that the extracts contribute to a substantial rise in antioxidant potential, immune parameters, and productivity in general [91]. What is more, the protective impact on oxidative stress and infectious diseases is also well-known with regard to the antioxidant effects of plant bioactive compounds [92].

### 3.4. Immune Regulation and Inflammatory Modulation by Phytogenic Additives

Recent studies have focused on the ability of specific phytogenic compounds to modulate inflammatory processes. Plant-based bioactive compounds may regulate immune signaling cascades, such as the *NF-κB* pathway, and modulate cytokine responses, including interleukins such as *IL-1β* and *IL-6* and tumor necrosis factor alpha (*TNF-α*), while potentially supporting mucosal T-cell differentiation and local immunoglobulin production [93]. Numerous phytochemicals increase the effect of immune cells, stimulate the production of antibodies, and inhibit the overproduction of inflammatory reactions [94,95]. The phytogenic additives aid in the maintenance of immune balance and elevated resistance to infectious challenges in poultry through these mechanisms. Some of the herbal derivatives also control the expression of cytokines, and they also decrease oxidative inflammation, which promotes physiological stability [66]. Extensive studies of the effects of phytogenic additives in livestock systems demonstrate their contribution to the immune competence and health of the animal [8]. The immune response, antioxidant defenses, and disease resistance of poultry production have also been reported to be enhanced with the help of nutrition strategies that include the use of phytogenic additives [96,97,98].

### 3.5. Intestinal Barrier Enhancement and Pathogen Control Mechanisms of Phytogenic Additives

The intestinal barrier is vital for nutrient absorption and for preventing the entry of pathogens. Phytogenic feed supplements may modify intestinal morphology by enhancing villus height, optimizing crypt depth, and improving epithelial cell integrity [99,100]. These structural changes may increase the absorptive surface area and thereby improve nutrient assimilation. Furthermore, bioactive plant compounds may strengthen intestinal barrier function by upregulating the expression of specific tight junction proteins, such as *Claudin-1, Occludin*, and *ZO-1*, through modulation of the *NF-κB* and *MAPK* signaling pathways, thereby potentially reducing intestinal permeability and microbial invasion [101,102].

In addition to supporting intestinal barrier function, phytogenic compounds may exhibit antimicrobial activity against bacterial and protozoal pathogens in poultry production systems [92]. Mechanistically, phytochemicals such as EOs, tannins, and alkaloids possess lipophilic properties that may allow them to partition into the lipid bilayer of microbial cell membranes. This interaction can disrupt membrane integrity, inhibit critical enzymatic systems, and alter metabolic pathways necessary for pathogen survival [103]. In protozoal infections, particularly those caused by *Eimeria* spp., specific phytogenic compounds may impair sporulation, reduce oocyst viability, and disrupt intracellular development, thereby potentially limiting disease severity and supporting anticoccidial activity [104]. However, these anticoccidial effects require careful attribution, as efficacy varies substantially depending on the specific botanical compound used, such as *Artemisia* extracts or oregano EOs, and the experimental challenge model. By suppressing the proliferation of pathogenic microorganisms and parasites in the digestive tract, phytogenic supplementation may contribute to maintaining a balanced intestinal microbiota and supporting the overall health and productivity of the flock [105,106].

## 4. Phytogenic Contributions to Poultry Productivity and Product Quality

The interest in PFAs in poultry nutrition has increased significantly due to their ability to enhance productivity, well-being conditions, and quality of the final product. These natural products affect various physiological functions such as digestion, metabolism, immune cellular functions, and balance of microbes [107]. This is because of their multifunctional nature, which enables them to aid growth performance and at the same time improve the quality and safety of poultry products. The key production and health effects of phytogenic supplementation that are evident in the poultry diets are summarized in Table 2. However, a critical synthesis of these outcomes reveals significant heterogeneity, with numerous studies reporting null or inconsistent findings. It is important to note that under optimal management and health conditions, where birds are not exposed to significant environmental or pathogenic stress, PFAs may fail to significantly improve body weight gain (BWG) or feed conversion ratio (FCR). Furthermore, inappropriate inclusion rates may result in negative performance outcomes due to dose-dependent toxicity or disruption of gut homeostasis. Despite this variability, these complex advantages have made phytogenic compounds a significant nutritional approach to enhance productivity and sustainability in contemporary poultry production systems.

### 4.1. Phytogenic Enhancement of Growth Performance and Feed Utilization

PFAs may influence growth performance through effects on digestive function, nutrient metabolism, intestinal microbial ecology, and oxidative status [4,111,112,113,114,115,116,117]. Studies involving cinnamon, turmeric, and other botanical preparations indicate that these responses may vary according to the formulation, composition, and dose of the additive [112,113,114]. Advanced delivery systems, including chitosan/alginate matrices and lipid-based carriers, are being investigated for their ability to protect volatile or heat-sensitive compounds during feed processing and to modify their release within the gastrointestinal tract [115,116]. However, improved protection during processing or enhanced intestinal delivery should not be interpreted automatically as evidence of increased systemic bioavailability.

### 4.2. Influence of Phytogenic Additives on Carcass Traits and Meat Quality

PFAs may influence carcass characteristics and meat quality through their effects on lipid metabolism and oxidative stability [118]. Recent experimental evidence in Japanese quail demonstrated that dietary garlic powder supplementation at 0.5%, 1%, and 2% influenced growth performance and selected meat-quality parameters, with the greatest growth response observed at the 1% inclusion level [37]. Plant-derived antioxidants may also help to reduce oxidative damage; however, the magnitude of their effects depends on factors such as botanical composition and dose [119,120,121,122]. In the context of Kuwait and the wider Gulf region, botanicals including *Artemisia herba-alba*, *Ziziphus spina-christi*, and *Moringa peregrina* have been investigated for their phytochemical composition and antioxidant properties [123,124,125]. These findings provide a regional rationale for further research but should not be interpreted as conclusive evidence that these plants improve poultry performance, because direct and well-controlled poultry-feeding studies involving these specific native botanicals remain limited. Accordingly, their potential is presented as a research priority requiring targeted in vivo validation, as discussed in Section 8.

### 4.3. Reproductive Performance and Egg Quality Responses to Phytogenic Supplementation

By increasing metabolic balance and nutrient utilization in laying birds, phytogenic compounds have the ability to increase reproductive performance and egg production [126]. Bioactive molecules are usually found in herbal feed additives and support reproductive physiology, enhance hormonal equilibrium, and boost antioxidant defenses in reproductive tissue [127]. Aloe vera is one of the most extensively tested herbal feed additives due to its high bioactive content and useful impact on livestock productivity and metabolic status [128]. Moreover, natural plant-based growth promoters were found to enhance gut health and digestive performance, and indirectly increase egg production and egg quality characteristics in poultry [129]. Some kinds of phytogenic compounds like pomegranate extracts have also been shown to have a positive impact on physiological health and productivity in poultry systems [130].

### 4.4. Mitigation of Heat Stress and Environmental Challenges

Other environmental stress factors, like high temperature, have a negative impact on the productivity of poultry by interfering with metabolic processes and physiological functions [131]. Importantly, evaluating native phytogenic plants from arid environments, such as the Gulf region, should not be viewed merely as a geographic focus. Rather, these desert-adapted botanicals may serve as useful scientific models for identifying resilient antioxidant compounds with potential applications for mitigating heat stress under diverse production conditions [132,133]. To accurately bridge experimental evidence with extreme production conditions, studies should evaluate specific temperature-humidity indices (THI), duration of heat exposure, and physiological indicators such as serum corticosterone levels, heterophil-to-lymphocyte ratios, oxidative stress biomarkers, and flock mortality [134]. Early nutritional management can reduce heat-stress-related physiological disruption in broilers and improve production stability [135]. Combinations of probiotics and phytogenic ingredients can improve antioxidant status, cecal microbiota, and growth performance under heat stress [136]. Antioxidant supplementation combined with feed-management strategies can further improve thermotolerance under heat stress [137]. Phytogenic feed supplements have demonstrated the ability to reduce such negative impacts since most of the plant components have antioxidant, anti-inflammatory, and immunomodulatory effects. These compounds contribute to stabilization of physiological balance associated with stressful conditions because they minimize oxidative damage and stabilize metabolic processes [138]. Resistance to environmental stress and sustainability of productivity in poultry production have been reported to be enhanced by nutritional interventions in which phytogenic additives are used [139]. Moreover, regulating gut health through phytogenic compounds promotes better physiological adaptation and overall health, even under stressful environmental conditions [107]. The experimental assessments of phytogenic feed supplements have also shown an increase in broiler performance when the additives are added to the poultry feed [118]. Furthermore, in hot-climate poultry systems, the effectiveness of nutritional interventions should also be interpreted in relation to drinking water quality and management, since the microbiological and physicochemical characteristics of drinking water can influence gut health, nutrient utilization, and productive performance [140].

### 4.5. Food Safety Enhancement and Pathogen Control Through Phytogenic Additives

Phytogenic feed supplements contribute positively to enhancing food safety by inhibiting the growth of microbes in poultry feed [53]. A large number of natural products of plants have antimicrobial properties that prevent the growth of pathogenic microorganisms within the digestive tract [141]. However, reduced intestinal pathogen abundance should not automatically be equated with improved food safety. While these additives may modulate the gut microbiota of live birds, demonstrating enhanced food safety requires evidence of reduced levels of foodborne pathogens, such as *Salmonella* or *Campylobacter*, at slaughter or on edible poultry products [142]. Aromatic plants and EOs have been widely researched in terms of their power to inhibit microbial disease and promote microbial safety in poultry production [143]. In the same way, oil-based feed additives that are regarded as essential have proven antimicrobial properties that may be used to suppress detrimental bacteria and enhance the health of animals [61]. Other feed additives with prebiotic-like characteristics have demonstrated beneficial effects on productivity and may help reduce dependence on AGPs [144].

## 5. Technological Innovations and Advanced Formulation Strategies

**Figure 2:**
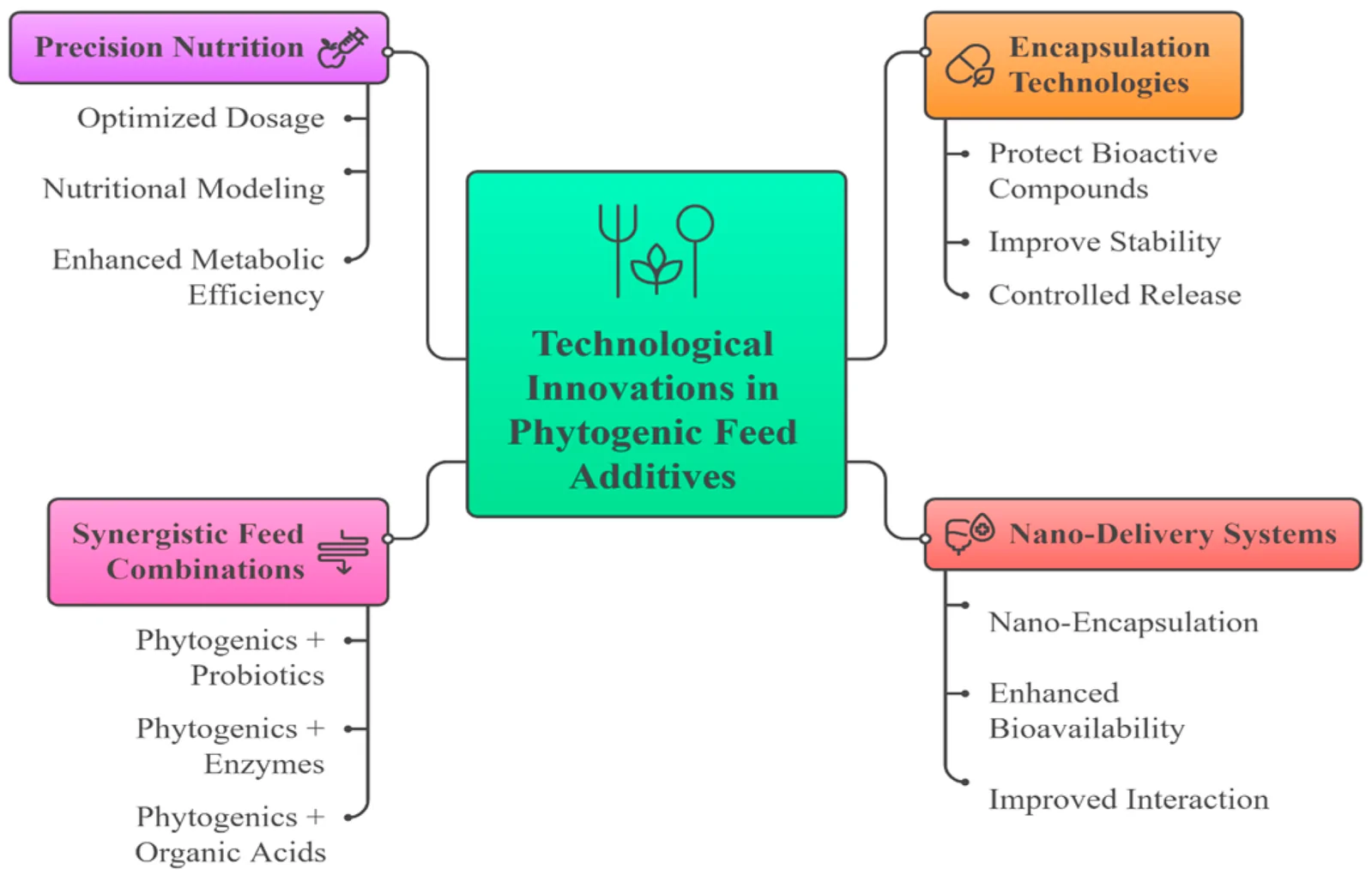
Technological innovations in PFAs for poultry nutrition, highlighting encapsulation technologies and nano-delivery systems to enhance stability, protection, and bioavailability of bioactive compounds, along with complementary feed combinations and precision nutrition strategies that optimize dosage, improve metabolic efficiency, and support sustainable poultry production with reduced antibiotic reliance [145,146,147,148].

Recent developments in poultry nutrition have focused on improving the performance and stability of PFAs through novel technological approaches. These strategies include improved delivery systems, advanced formulations, and complementary integration with other functional feed components. The key technological strategies that may improve the application and efficacy of phytogenic additives in poultry feed are shown in Figure 2.

### 5.1. Encapsulation Technologies

The development of encapsulation technologies has become a good means to enhance the stability and delivery of the PFAs in poultry diets and enhance their performance [149]. Most compounds present in plants are environmentally sensitive to heat, oxygen, and moisture, and these may inhibit their functional activity during feed processing. Encapsulation methods keep such bioactive compounds in protective matrices that release them gradually in the gastrointestinal tract [145]. The phytogenic compounds delivered by controlled-release systems are delivered to specific locations in the digestive system, therefore maximizing their biological activities in gut health and metabolism [150]. The prior research emphasizes the fact that phytogenic compounds in the nutrition of poultry can be enhanced in effectiveness and stability with the help of advanced feed technologies, including microencapsulation [151]. This helps to increase the functional performance of plant-based additives and aid their use in the current production systems of poultry [152].

### 5.2. Nano-Delivery System

Nanotechnology and microencapsulation are being investigated as strategies to improve the stability and site-specific intestinal delivery of phytogenic compounds. In poultry, the most robust quantitative evidence currently concerns intestinal delivery rather than systemic bioavailability. For example, Zhang et al. developed alginate-whey protein microparticles containing carvacrol, achieving encapsulation efficiencies of at least 98%. In chickens, less than approximately 3% of unencapsulated carvacrol was detected beyond the gizzard, whereas up to approximately 20% was detected in the ileum following administration of the optimized microparticles. The microparticles also maintained elevated intestinal carvacrol concentrations for approximately 3 h [153].

Accordingly, this review distinguishes among intestinal delivery, bioaccessibility, and bioavailability and avoids treating production performance as a surrogate measure of bioavailability. Other nano-delivery studies have reported improvements in digestibility or feed efficiency [154,155]. However, these findings should be described as biological or production responses unless supported by pharmacokinetic data or measurements of tissue exposure. The commercial translation of these technologies will also require careful consideration of manufacturing scalability, stability during feed pelleting, release kinetics, carrier safety, and regulatory requirements [116,156].

### 5.3. Complementary Feed Formulations

The use of PFAs together with other functional feed additives has also attracted interest as a means of increasing the efficiency of plant-based feed additives. Probiotics, enzymes, and organic acids can be used in complementary formulations to enhance the efficiency of the digestive system, microbial balance, and immune system in birds [147]. To illustrate potential interaction mechanisms, lipophilic phytogenics may disrupt pathogenic bacterial cell membranes and thereby influence the competitive establishment of probiotic strains, such as *Bacillus subtilis* [157]. Concurrently, combining PFAs with specific exogenous enzymes, such as xylanase, may reduce digesta viscosity and potentially improve the availability of phytogenic active compounds within the intestinal environment, thereby supporting nutrient utilization [158]. These combinations enable various feed additives to balance each other in their biological activities, resulting in better gut health and nutrient utilization. The combination of probiotics and phytogenic compounds can stabilize the microbiota of the intestines and avoid harmful bacteria, improving the activity of the intestines [107]. The research on the alternative feed additives stresses that the combination of phytogenic compounds with probiotics and other useful ingredients can contribute to the maximal optimization of poultry gut health and performance [101]. These combined nutritional plans are a promising solution to the enhancement of productivity and the elimination of dependence on AGPs [159].

### 5.4. Precision Nutrition Strategies

Precision nutrition concepts are also being used in the feeding of poultry to maximize the dosage and efficacy of PFAs [21]. It is necessary to determine the proper levels of inclusion since over- or under-supplementation will decrease the efficacy of such substances. Crucially, dose optimization must be explicitly tied to measurable active compounds rather than merely the total mass of the botanical preparation [160]. For instance, two turmeric extracts administered at the same inclusion rate may exhibit substantially different phytochemical profiles [161]. Current feeding strategies combine nutritional modeling, monitoring of metabolic systems, and manipulated feeding programs so that phytogenic additives are provided at the most appropriate dosages [162]. Moving forward, a clear framework for implementation in modern poultry systems may rely on advanced data-driven approaches. Specifically, such a framework could integrate real-time environmental sensors, including monitoring of temperature and humidity fluctuations, with omics-derived health biomarkers from the flock. These continuous data streams could be processed using artificial intelligence (AI) algorithms to dynamically adjust the dietary inclusion rates of specific phytogenic blends according to environmental conditions and physiological indicators of the birds [163]. This can be done through these strategies to maximize the physiological benefits and to minimize the non-essential feed costs. Recent developments in the field of poultry nutrition indicate that well-balanced phytogenic supplementation may positively influence the production systems of poultry in terms of improved metabolic efficiency, development, and sexual health [148]. Also, studies of plant-based feed additives emphasize their possible use in improving growth and nutrient uptake, and metabolic signaling pathways in broiler chickens [114]. The recent literature highlights the growing role of phytogenic additives as nutraceutical components that can be used to maintain the health, productivity, and sustainability of poultry in the modern production facilities [164]. On top of that, the use of phytogenic substances in combination with other feed additives was suggested as one of the approaches to help restore the gut microbial balance and enhance the overall health of poultry [79]. Reviews have also noted beneficial aspects of medicinal plant supplementation, which include nutritional benefits and their possible application in the production of poultry in a sustainable manner [164]. Further studies on new formulations of feeds are thus critical in ensuring that the advantages of phytogenic compounds in the poultry diets are maximized [165].

## 6. Decoding Phytogenic Effects Through Omics Technologies

Current advances in the molecular biology sector have introduced the instruments of the omics that would enable a clearer understanding of the impact of PFAs on poultry physiology, metabolic activity, and digestive well-being. The techniques help scientists to investigate complex biological interactions of dietary compounds, host metabolism, and intestinal microbiota. However, it is important to recognize that while omics approaches excel at revealing associations and identifying candidate pathways, establishing definitive causal mechanisms generally requires subsequent targeted biological validation. A genomic, microbial, and metabolic combination of information would assist scientists in gaining a better understanding of how the phytogenic compounds can be utilized to enhance the performance and health of the poultry. The most important omics techniques involved in the research of PFAs are listed in Table 3. This table shows that the omics technologies may provide useful insights into how phytogenic compounds affect biological systems at different levels, including gene regulation, the dynamics of the microorganisms, and metabolism. These integrative systems work well in identifying functional mechanisms so as to guarantee improved productivity and health in poultry.

### 6.1. Gene–Nutrient Interactions and Nutrigenomic Responses

In biology, nutrigenomics examines how dietary constituents influence metabolism and gene expression. The phytogenic compounds of herbs, spices, and EOs are able to interact with the cellular signaling pathways of poultry nutrition, which leads to alterations in the transcriptional activity of immune responses, oxidative balance, and nutrient utilization [173]. The information on responding genes and networks under the impact of phytogenic supplementation can be obtained with the help of transcriptomic methods. It is also demonstrated that bioactive compounds with a vegetal origin can enhance the expression of antioxidant-related genes and strengthen the intestinal barrier, which can be translated into enhanced physiological health and productivity in broiler chickens [66]. In addition, transcriptome analyses have also found ways in which feed supplements may be applied to manipulate the genes related to the growth rate, immunity, and metabolic acclimatization, which can be utilized in the poultry feeding programs by developing effective alternatives to antibiotics [166].

### 6.2. Microbiome Dynamics and Host Microbe Interactions

Microbiome and metagenomic approaches provide powerful tools to better understand how PFAs influence the gut microbial communities in poultry [174]. To achieve greater mechanistic depth, modern microbiomics should advance beyond standard 16S rRNA sequencing, which primarily characterizes microbial taxonomic composition, toward shotgun metagenomics, which can provide information on microbial functional genes and pathways, including carbohydrate-active enzymes [175]. These techniques enable comprehensive identification of microbial populations and their functional genes, offering deeper insights into microbial fermentation processes and host–microbe interactions [176]. Concurrently, evaluation of the host response increasingly relies on modern transcriptomic approaches. By transitioning from traditional microarrays to high-throughput RNA sequencing (RNA-seq), researchers can more comprehensively characterize changes in gene expression, including the downregulation of pro-inflammatory signaling pathways involving genes such as *IL-1β* and *TNF-α* within the *NF-κB* cascade and the upregulation of antioxidant-related genes [177,178]. In most instances, phytogenic compounds possess antimicrobial and modulatory properties, which lead to the suppression of pathogenic microorganisms and favor prokaryotic microbiomes. This form of microbial modulation assists in improved digestion of nutrients, immune responses, as well as intestinal protection. The research examining the relationships between microbes and their diets has shown that the phytogenic additives can potentially change the metabolic activities and fermentations in microbes, thus affecting the overall digestive efficiency [179]. Also, the promising qualities of plant extracts, especially EOs, have demonstrated usefulness in regulating the number of microbes in the gut and improving the stability of the intestine in poultry production systems [116].

### 6.3. Metabolomics Reprogramming and Biomarker Identification

Metabolomics has attracted interest in the mass examination of the metabolites produced amid biological processes and offers information that is useful in the physiology of the animal for dietary intervention [71]. Modern metabolomic profiling requires advanced analytical platforms to characterize complex metabolic changes. Liquid chromatography mass spectrometry (LC-MS) can be used to analyze a broad range of polar metabolites in blood or tissues, whereas gas chromatography mass spectrometry (GC-MS) is commonly used to quantify volatile and derivatizable metabolites, including short-chain fatty acids such as butyrate in the ceca [180]. Metabolomic analysis of poultry has been assigned the role of showing how the phytogenic compounds affect immunological, energy metabolic, and oxidative defense metabolic processes [118]. By analyzing the metabolites in the tissues, blood, and intestines, researchers can establish some biomarkers that can be associated with improved health status and production performance [181]. These metabolic indicators could be mentioned to elaborate on the functionalities of PFAs in poultry [118]. The metabolomic studies are also applicable as far as the evaluation of the preventative strategies in the diets is concerned, which can assist with the preservation of disease resistance and physiological stability within the poultry systems [182]. Besides, one can consider metabolite-based measurements to study the response of detoxification and assess phytogenic compounds that ameliorate feed contaminants resulting in mycotoxin poisoning [183].

### 6.4. System-Level Integration Through Multi-Omics Modeling

Multi-omics approaches are integrative approaches that entail the integration of information on genomes, transcriptomes, microbiomes, and metabolomes to provide an entire picture of biological responses to phytogenic compounds in diets. Systems biology modeling enables researchers to investigate the interdependence among nutrients, host metabolism, and microbial populations [184]. However, systems biology should not be presented merely as the simple combination of several datasets. Successful integration of microbiome, transcriptome, and metabolome data requires rigorous experimental design, adequate biological replication, appropriate statistical correction for multiple testing, careful management of batch effects, and independent experimental validation [185]. Integrating these data layers into a coherent multi-omics framework requires appropriate bioinformatics and statistical approaches, such as weighted gene co-expression network analysis (WGCNA) and multi-block partial least squares methods [186]. A potential example of this integration would involve associating an increase in *Lactobacillus* abundance, determined through shotgun metagenomics, with elevated cecal butyrate concentrations measured by GC-MS and changes in mucosal tight junction gene expression measured by RNA-seq [187]. Such integrated analyses may help identify regulatory relationships associated with growth, immune function, and stress tolerance in poultry. However, routine application of these technologies faces major limitations, including the high cost of comprehensive omics platforms, substantial technical and bioinformatic challenges in data processing, and limited applicability for rapid on-farm decision-making in commercial poultry systems [184]. The techniques are gaining popularity in the evaluation of the functional activity of feed additives in various environmental and production conditions. The systems-level research can also assist in developing accurate feeding plans in a proper manner that is able to optimize poultry health and production [188]. In addition, the use of phytogenic embryonic nutritional supplementation has been observed to alter metabolic and immune systems during adulthood, and other systems-based nutritional interventions hold promise in poultry production.

## 7. Safety and Regulatory Perspectives of Phytogenic Feed Additives

The use of PFAs in poultry nutrition must be done with great caution regarding safety, possible toxicological effects, and regulatory provisions. It is necessary to consider anti-nutritional factors, accumulation of residues, and international regulatory standards in order to ensure animal health and consumer protection. The scientific studies focus on the idea that the formulation and quality should be addressed to achieve the consistency of efficacy and safety of phytogenic compounds that are used in the livestock production systems [189]. This is done to make sure that the phytogenic compounds may be safely included in poultry feeding programs without compromising product quality and consumer safety. A One Health framework is essential when phytogenic feed resources are scaled up, because animal, human, and environmental safety are interconnected [190].

### 7.1. Toxicological Risks and Anti-Nutritional Constraints of Phytogenic Compounds

Although the phytogenic compounds have some functional advantages, some of them can have anti-nutritional effects when consumed in large amounts [191]. Alkaloids, tannins, and EOs are bioactive compounds that can interfere with digestive enzymes, nutrient intake, or metabolism when present above safe inclusion levels [8]. It is thus important to set up toxicity limits to prevent adverse physiological reactions in poultry. Specifically, excessive inclusion rates can lead to dose-dependent toxicity, substantial reductions in feed intake due to poor palatability, and undesirable interactions with basal dietary nutrients or concurrently administered medications. Furthermore, natural origin does not preclude the presence of environmental contaminants, such as heavy metals or mycotoxins, when botanical sourcing and quality control are inadequate [44]. The studies of the topic on EOs and plant extracts emphasize that their biological activity is highly concentration-dependent, with chemical composition and digestive system interaction [192]. Moreover, the evidence from controlled studies testing dietary additives with origins of plant-based products suggests that the right proportions of these substances can positively affect nutrient use and intestinal safety without injuring the metabolic performance of poultry [193].

### 7.2. Residue Transfer, Food Safety and Consumer Health Implications

Consumer safety should be handled well in case phytogenic additives are utilized in the diets of poultry. Even though the majority of the plant-derived compounds are quickly excreted, it is important to trace their future presence in the edible products in the form of meat and eggs. The scientific research shows that phytogenic supplementation may improve immunogenesis and body resilience in broiler chickens. While specific studies evaluating targeted extracts [194] have reported rapid clearance and negligible harmful residues in edible tissues, a class-wide assumption of residue safety cannot be made given the substantial chemical heterogeneity of PFAs. Therefore, safety profiling should be established on a compound-specific basis. Moreover, the extensive studies of nutritional plant extracts have shown the enhancement of the growth performance, gut microbiota profiles, and blood biochemical indicators, which posits that such supplements can be used to enhance the health of animals without affecting the safety of the product to the human body [195].

### 7.3. Regulatory Harmonization and Global Standardization of Phytogenic Additives

The production of PFAs is controlled by the international and national bodies to guarantee product safety, quality, and effectiveness. However, classification and authorization requirements differ significantly between jurisdictions. For instance, the European Food Safety Authority (EFSA) applies distinct regulatory frameworks for the authorization of zootechnical and sensory feed additives, whereas in the United States, the Food and Drug Administration (FDA) regulates relevant substances through applicable regulatory pathways, which may include Generally Recognized as Safe (GRAS) provisions or approval as specific food additives [196,197]. Such regulatory frameworks normally necessitate scientific interaction with chemical characterization, toxicological examination, and functional execution before authorization is given for the application of animal feeding. The recent advances in the field of feed additive technology, such as innovative delivery models of phytogenic substances, have compromised the issue of regulatory control to ensure the stability and safety of products in the area of non-ruminant nutrition [198]. Further, the phytogenic compound reviews show that consistent procedures of evaluation and quality control are required to guarantee the same biological effect and safe addition to poultry feeding programs across the globe [199].

## 8. Current Limitations and Research Gaps

Lack of methodological homogeneity remains a major limitation in PFA research. Trials frequently differ in bird genetics, basal diet composition, phytogenic source and composition, extraction method, dose, formulation, and environmental conditions, which complicates direct comparison of results. This issue is especially relevant when interpreting findings for Kuwait and other Gulf production systems, where high ambient temperatures and arid conditions interact with feed and water constraints. Rather than assuming that results obtained under temperate conditions will transfer directly, future studies should adopt standardized experimental frameworks and report environmental variables, phytochemical composition, inclusion rates, and performance endpoints in sufficient detail to enable reproducibility and region-specific validation [110,200,201].

Phytogenic compounds also exhibit substantial variability in biological activity arising from differences in plant species, chemotype, extraction method, and inclusion level [202]. Dose-response relationships should therefore be established on the basis of the measured concentration of relevant active constituents rather than the mass of the botanical product alone. In Kuwait and other Gulf systems, heat stress and variability in feed ingredients may further modify responses, underscoring the need for controlled, regionally relevant trials that quantify both efficacy and safety instead of assuming a universal optimal dose [110].

Most PFA studies have examined only short- or medium-term production outcomes, while evidence spanning complete production cycles remains limited. The appropriate duration of long-term evaluation differs among broilers, layers, and breeders; studies should therefore define the biological period a priori and assess the persistence of production, immune, microbiome, and safety outcomes. In hot-climate systems, longitudinal trials should also record thermal conditions and other environmental covariates so that time-dependent interactions can be distinguished from treatment effects [203]. Studies conducted in Kuwait and the wider Gulf region can provide valuable validation under these conditions, but their findings should be regarded as context-specific until they are replicated across other production systems.

A further critical limitation is inconsistency in statistical and experimental design. Future PFA studies should clearly identify the experimental unit, provide sample-size justification, employ randomization and blinding where feasible, apply appropriate dose-response models, report effect sizes with confidence intervals, and describe the handling of mortality and missing data. Multiple-testing adjustment should be applied when several endpoints are evaluated [200]. These principles are particularly important for regional validation studies, in which limited farm-level replication can otherwise be mistaken for robust generalizability.

A persistent challenge in phytogenic research is the translation of laboratory findings into practical commercial application. Controlled experimental studies typically involve small flock sizes and highly controlled environmental conditions that differ markedly from those of large-scale commercial poultry production. In Gulf countries such as Kuwait, farms commonly experience heat stress, fluctuating water quality, and intensive housing systems, all of which can influence additive performance [201]. Consequently, results obtained under experimental conditions may not necessarily be reproduced in commercial settings. To bridge this gap, field-scale trials conducted under practical farm conditions are required. Commercial validation should extend beyond biological performance to encompass economic return on investment, additive cost, product consistency, feed-processing stability (including survival during pelleting), and interactions between PFAs and intensive commercial management practices.

## 9. Future Directions in Phytogenic Research

Future research should place greater emphasis on phytogenic preparations that remain effective under arid and semi-arid production conditions. Within the Kuwait and Gulf-region perspective of this review, heat stress is a major practical constraint that can reduce feed intake, impair immunity, and lower productivity [138]. Native or regionally available botanicals may serve as useful research models for identifying antioxidant or thermoprotective compounds; however, local origin alone does not constitute evidence of efficacy or sustainability. Candidate materials should advance from phytochemical screening to replicated in vivo poultry trials that employ standardized extracts, defined active compounds, dose-response characterization, safety assessment, and commercial-scale validation. Research should also evaluate seasonal availability, extraction yield, ecological consequences of harvesting, competing human uses, and economic scalability. Such regional work can generate hypotheses and candidate materials of broader relevance to poultry systems facing increasing climate-related stress, provided the distinction between regional evidence and general poultry evidence remains explicit [204,205,206].

The integration of AI and predictive analytics may assist in optimizing PFA selection and inclusion rates, yet reliable application depends on standardized phytochemical data, accurate diet formulations, environmental measurements, longitudinal bird- or flock-level outcomes, and independent validation. In Kuwait and other Gulf production systems, models could incorporate seasonal temperature and humidity data, but region-specific performance must be demonstrated before results are extrapolated more widely. Standardization of botanical preparations, robust dose–response trials, commercial heat-stress validation, and reproducible biomarkers remain essential prerequisites for useful predictive models [207].

PFAs may support more sustainable poultry production when validated improvements in nutrient utilization, gut health, and feed efficiency translate into measurable production gains. In Kuwait and the wider Gulf region, locally available plant materials and agro-industrial by-products may be examined as candidate feed resources; their sustainability, however, should be assessed based on feed value, phytochemical consistency, land and water requirements, competing uses, safety, and life-cycle considerations rather than geographic origin alone. Complementary approaches such as resource recycling and alternative feed ingredients may contribute to broader sustainability objectives, but each intervention requires direct evidence obtained under practical poultry-production conditions [190,206].

Commercial implementation of PFAs in Kuwait and neighbouring Gulf countries will require coordinated efforts among researchers, feed manufacturers, producers, and regulators. Pilot-scale studies should quantify biological efficacy together with product consistency, processing stability, economic return, and safety under practical farm conditions. These regional investigations can help close the translational gap between experimental findings and commercial use while providing a clearly defined context for the present review, without implying that Kuwait-specific results are universally applicable.

## 10. Conclusions

PFAs can form useful components of integrated antibiotic-reduction strategies in poultry nutrition, yet they should not be regarded as generic one-to-one replacements for conventional AGPs. Reported effects on growth performance, gut health, immune function, oxidative status, and product quality remain heterogeneous and are strongly influenced by botanical composition, dose, formulation, diet, bird type, and environmental conditions. Recent advances in delivery technologies and omics approaches have improved mechanistic understanding, but substantial gaps persist in standardization, dose-response characterization, long-term validation, and direct measurement of intestinal delivery and bioavailability. The Kuwait and Gulf-region perspective developed in this review identifies heat stress and regional feed-resource constraints as relevant contexts for targeted validation rather than as a basis for generalizing local findings. Future research should integrate rigorous in vivo experiments, quantitative assessments of delivery and bioavailability, commercial-scale validation, and appropriately validated predictive tools. The overarching mechanistic framework is that PFAs function as context-dependent biological modulators whose efficacy depends on reproducible composition, optimized delivery, and interactions among host, microbiome, and environmental factors.

## Figures and Tables

**Table 1:** Forms and bioactive components of PFAs in poultry.

PFA Formulation Type	Active Phytochemical Compound(s)	Primary Target Mechanism	Physiological Target Pathway	Representative Dose/Study Type; Key Outcome and Evidence	Refs.
Essential Oils (e.g., oregano, thyme, cinnamon, clove)	Terpenoids/phenolics: carvacrol, thymol, cinnamaldehyde, eugenol	Direct disruption of pathogenic bacterial cell membranes (hydrophobic partitioning into the lipid bilayer, increased permeability, leakage of intracellular contents); modulation of ileal *TLR2*, *TNF-α*, *IL-1β* and tight-junction (*claudin-1*, *occludin*, *ZO-1*) gene expression	Bacterial membrane integrity; intestinal epithelial barrier and innate-immune signaling	Representative dose range: 60–600 mg/kg across controlled broiler studies. Study type: controlled in vivo broiler feeding/challenge trials. Outcome: antimicrobial, barrier, and performance-related effects vary by compound and dose.	[22,23,24,25]
Plant Extracts (e.g., green tea, neem, aloe vera)	Phenolics: polyphenols, flavonoids, tannins	Direct free-radical (ROS) scavenging, combined with up-regulation of the endogenous *Nrf2/Keap1-ARE* antioxidant pathway (Keap1, HMOX1, SOD1, CAT, NQO1)	*Nrf2/Keap1/ARE* signaling axis	Representative doses: 0.1% neem leaf aqueous extract and 0.1–0.2% aloe vera powder. Study type: controlled in vivo broiler feeding trials. Outcome: effects on growth, gut health, antioxidant status, and microbial populations are formulation- and dose-dependent.	[26,27,28,29,30]
Herbs & Rhizomes (e.g., garlic, ginger, turmeric)	Organosulfur compounds (allicin), gingerols, curcuminoids	Stimulation of endogenous digestive enzyme secretion; organosulfur-mediated antimicrobial activity	Pancreatic and digestive secretory function	Representative dose: 0.5–2.0% garlic powder in a 5-week Japanese quail feeding trial; other herbs/rhizomes use different inclusion levels. Study type: controlled feeding trials. Outcome: effects on digestibility, growth, immunity, and meat quality depend on botanical source and dose.	[31,32,33,34,35,36,37]
Oleoresins (e.g., paprika, capsicum)	Capsaicinoids, carotenoids	Modulation of lipid metabolism and oxidative stability in muscle tissue	Muscle lipid oxidative-stability pathways	Representative dose range: 100–150 mg/kg in controlled broiler studies; the 100 mg/kg study used a carvacrol/cinnamaldehyde/capsicum formulation, while a separate study tested 150 mg/kg capsicum oleoresin. Study type: controlled broiler feeding trials. Outcome: effects on feed efficiency, growth, and meat quality are formulation-specific.	[38,39,40,41,42,43]

**Table 2:** Effects of PFAs on poultry performance, health, and product quality.

Poultry Type & Phase	Phytogenic Source & Dietary Dose	Production Aspect	Quantitative Outcome (BWG/FCR/Other)	Ref.
Broilers (starter & finisher phases)	Nanoencapsulated rosemary essential oil (REO; 100 and 200 mg/kg)	Growth performance	At 200 mg/kg nanoencapsulated REO: BWG 1899 g/bird vs. 1742 g/bird control; FCR 1.62 vs. 1.77 (8.47% lower FCR).	[108]
Japanese quails (growth phase)	Garlic powder (0.5%, 1%, 2%)	Growth performance; meat quality	At week 3: BWG 83.25 g vs. 70.60 g control at 1% GP; FCR 1.97 vs. 2.23. Final BW: 281.63 g (1%) and 279.15 g (2%) vs. 272.96 g control.	[37]
Laying hens (65–73 week of age)	*Alquernat nebsui* L in drinking water (0.5 mL/L)	Egg production; feed efficiency	FCR 1.85 vs. 1.98 control; difference was numerical and not statistically significant (*p* > 0.05). Egg production rate improved in supplemented hens.	[109]
Broilers (42-day summer-stress trial)	Clove/eucalyptus EO blend (25 g/100 kg) and other PFA blends (10 g/100 kg)	Heat-stress mitigation; growth performance	Significant (*p* < 0.05) improvements in BWG and FCR were reported under summer stress; group means were not reproduced where not directly available in the accessible report.	[110]

**Table 3:** Major omics approaches used to evaluate PFAs in poultry nutrition.

Omics Approach	Key Application in Poultry Research	Functional Outcome	Refs.
Transcriptomics	Targeted qPCR/RNA-seq profiling of host gene expression in gut mucosa (duodenum/ileum/cecum) and, in reproductive-stage layers, in kidney, pancreas, follicle, and uterus	Up-regulation of Nrf2/Keap1-ARE pathway genes (Keap1, SOD1, HMOX1, CAT, NQO1) in broiler gut mucosa following phytogenic inclusion; modulation of tight-junction (claudin-1, occludin), TLR2, TNF-α and IL-1β expression under pathogen challenge; in laying hens, differential expression of antimicrobial-protein and bio-mineralization genes in pancreas/follicle/uterus	[24,29,166]
Metabolomics	LC-MS/GC-MS profiling of systemic (plasma) and local (cecal/intestinal) metabolic shifts	Identification of fermentation-derived and anti-inflammatory metabolites (e.g., short-chain fatty acids, immune-pathway metabolites) associated with phytogenic supplementation and coccidiosis-challenge models	[167,168,169]
Metagenomics/Microbiomics	16S rRNA and shotgun metagenomic sequencing to characterize microbial community structure and functional potential	Shifts in cecal microbial taxa and functional gene content (e.g., carbohydrate-active enzymes) associated with phytogenic versus antibiotic growth promoter feeding	[170,171,172]

## Data Availability

No new data were created or analyzed in this study. Data sharing is not applicable to this article.

## Abbreviations

PFAs	Phytogenic Feed Additives
AGPs	Antibiotic Growth Promoters
EOs	Essential Oils
AI	Artificial Intelligence
BWG	Body Weight Gain
FCR	Feed Conversion Ratio
